# The genome sequence of an ichneumonid wasp, *Heteropelma amictum *(Fabricius, 1775)

**DOI:** 10.12688/wellcomeopenres.20364.1

**Published:** 2023-11-20

**Authors:** Liam M. Crowley, Gavin R. Broad, University of Oxford and Wytham Woods Genome Acquisition Lab, Darwin Tree of Life Barcoding collective, Wellcome Sanger Institute Tree of Life programme, Wellcome Sanger Institute Scientific Operations: DNA Pipelines collective, Tree of Life Core Informatics collective, Darwin Tree of Life Consortium

**Affiliations:** 1 University of Oxford, Oxford, England, UK; 2 Natural History Museum, London, England, UK

**Keywords:** Heteropelma amictum, an ichneumonid wasp, genome sequence, chromosomal, Hymenoptera

## Abstract

We present a genome assembly from an individual female *Heteropelma amictum* (an ichneumonid wasp; Arthropoda; Insecta; Hymenoptera; Ichneumonidae). The genome sequence is 226.4 megabases in span. Most of the assembly is scaffolded into 10 chromosomal pseudomolecules. The mitochondrial genome has also been assembled and is 20.65 kilobases in length.

## Species taxonomy

Eukaryota; Metazoa; Eumetazoa; Bilateria; Protostomia; Ecdysozoa; Panarthropoda; Arthropoda; Mandibulata; Pancrustacea; Hexapoda; Insecta; Dicondylia; Pterygota; Neoptera; Endopterygota; Hymenoptera; Apocrita; Ichneumonoidea; Ichneumonidae; Anomaloninae; Gravenhorstiini; *Heteropelma*, *Heteropelma amictum* (Fabricius, 1775) (NCBI:txid494771).

## Background

*Heteropelma amictum* is one of our more conspicuous ichneumonid wasps in late summer and autumn, often seen in rather slow flight over bushes and shrubs with its clear yellow extremities nearly glowing. The red metasoma (the apparent abdomen) and yellow hind tarsi and antennae contrast strongly with the black mesosoma (thorax plus the first abdominal segment). Various species of the subfamily Anomaloninae share a very similar body shape and basic colour pattern but most (in Europe at least) are smaller and lack the particularly bright end to the antenna. The wing venation of *Heteropelma* species is relatively distinctive (see Gauld, 1976; Gauld & Mitchell, 1977). Males of *H. amictum* are seen more frequently, presumably searching for females, and at close range can be recognised by the strongly widened second hind tarsal segment. Identification of *H. amictum* is relatively straightforward using Gauld and Mitchell (1977) or Pénigot (2021), with the latter being more reliable for the subfamily Anomaloninae as a whole. The species has a very wide range, from most of Britain across Europe and Asia to Indonesia ( Gauld, 1976).

Found in various habitats, but particularly more open areas with shrubs, *H. amictum* has been reared from a variety of medium-sized caterpillars of the families Erebidae and Noctuidae ( Gauld & Mitchell, 1977). As with other Anomaloninae, the female oviposits in the host when it is a larva, with the wasp larva completing its development in the host pupa. All anomalonines of known biology are solitary parasitoids and the long legs and petiolate metasoma seem to be used to jab the caterpillar with the ovipositor from a distance, with the metasoma swung beneath the wasp’s body. How anomalonine larvae survive within the bodies of a range of host species is unknown, but there are reports that the larvae of at least some species might be protected within a trophamnion, a layer of cells surrounding the early instar larvae ( Rosenberg, 1934; Tothill, 1922). This is the first published genome for a species of Anomaloninae, and the growing body of genomes for the clade of ophioniform ichneumonid wasps should help us understand some of the innovations which have enabled this massive radiation of endoparasitoid koinobiont parasitioid wasps, i.e., wasps with larvae which develop within the active larvae of their hosts.

## Genome sequence report

**Figure 1.**
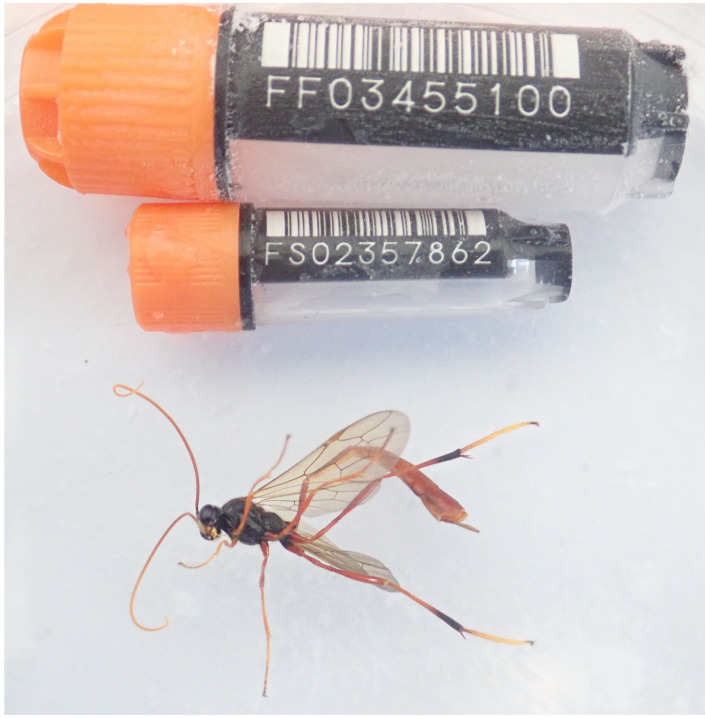
Photograph of the *Heteropelma amictum* (iyHetAmic1) specimen used for genome sequencing.

The genome was sequenced from one female *Heteropelma amictum* ( Figure 1) collected from Wytham Woods, Oxfordshire, UK (51.76, –1.33). A total of 101-fold coverage in Pacific Biosciences single-molecule HiFi long reads was generated. Primary assembly contigs were scaffolded with chromosome conformation Hi-C data. Manual assembly curation corrected 142 missing joins or mis-joins and removed one haplotypic duplication, reducing the scaffold number by 75.36%, and increasing the scaffold N50 by 83.43%.

**Figure 2.**
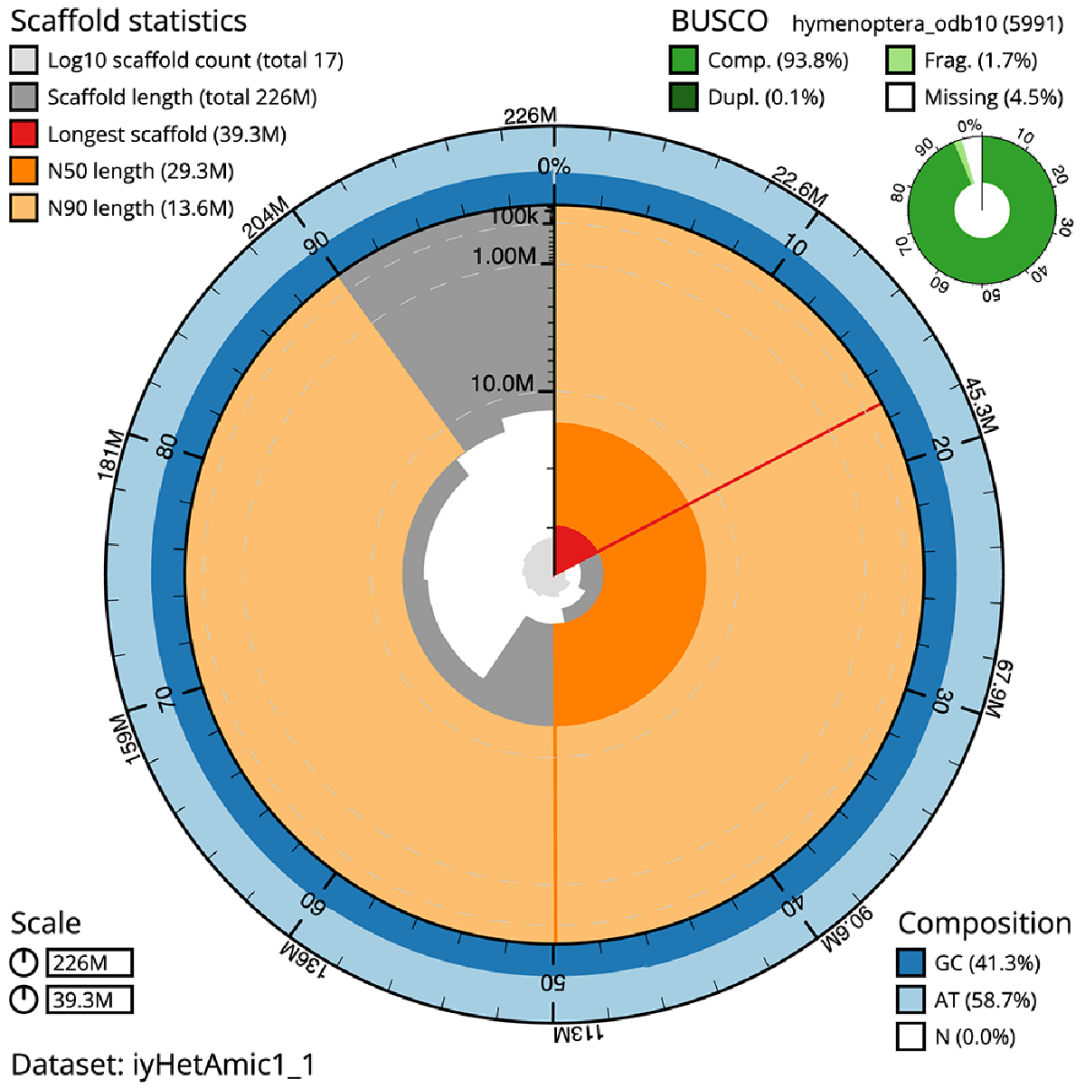
Genome assembly of *Heteropelma amictum*, iyHetAmic1.1: metrics. The BlobToolKit Snailplot shows N50 metrics and BUSCO gene completeness. The main plot is divided into 1,000 size-ordered bins around the circumference with each bin representing 0.1% of the 226,445,852 bp assembly. The distribution of scaffold lengths is shown in dark grey with the plot radius scaled to the longest scaffold present in the assembly (39,331,219 bp, shown in red). Orange and pale-orange arcs show the N50 and N90 scaffold lengths (29,282,975 and 13,633,056 bp), respectively. The pale grey spiral shows the cumulative scaffold count on a log scale with white scale lines showing successive orders of magnitude. The blue and pale-blue area around the outside of the plot shows the distribution of GC, AT and N percentages in the same bins as the inner plot. A summary of complete, fragmented, duplicated and missing BUSCO genes in the hymenoptera_odb10 set is shown in the top right. An interactive version of this figure is available at https://blobtoolkit.genomehubs.org/view/Heteropelma%20amictum/dataset/iyHetAmic1_1/snail.

**Figure 3.**
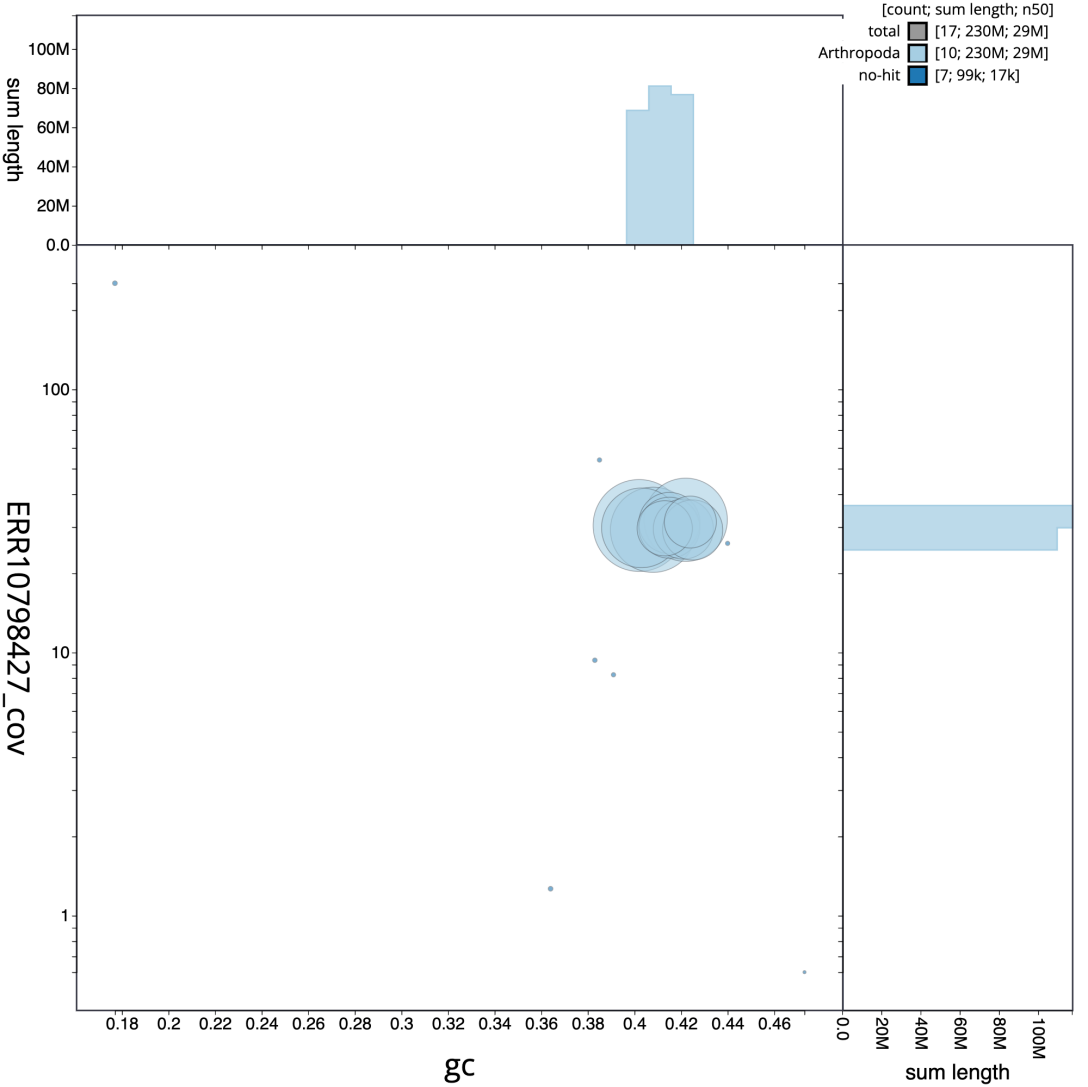
Genome assembly of *Heteropelma amictum*, iyHetAmic1.1: BlobToolKit GC-coverage plot. Scaffolds are coloured by phylum. Circles are sized in proportion to scaffold length. Histograms show the distribution of scaffold length sum along each axis. An interactive version of this figure is available at https://blobtoolkit.genomehubs.org/view/Heteropelma%20amictum/dataset/iyHetAmic1_1/blob.

**Figure 4.**
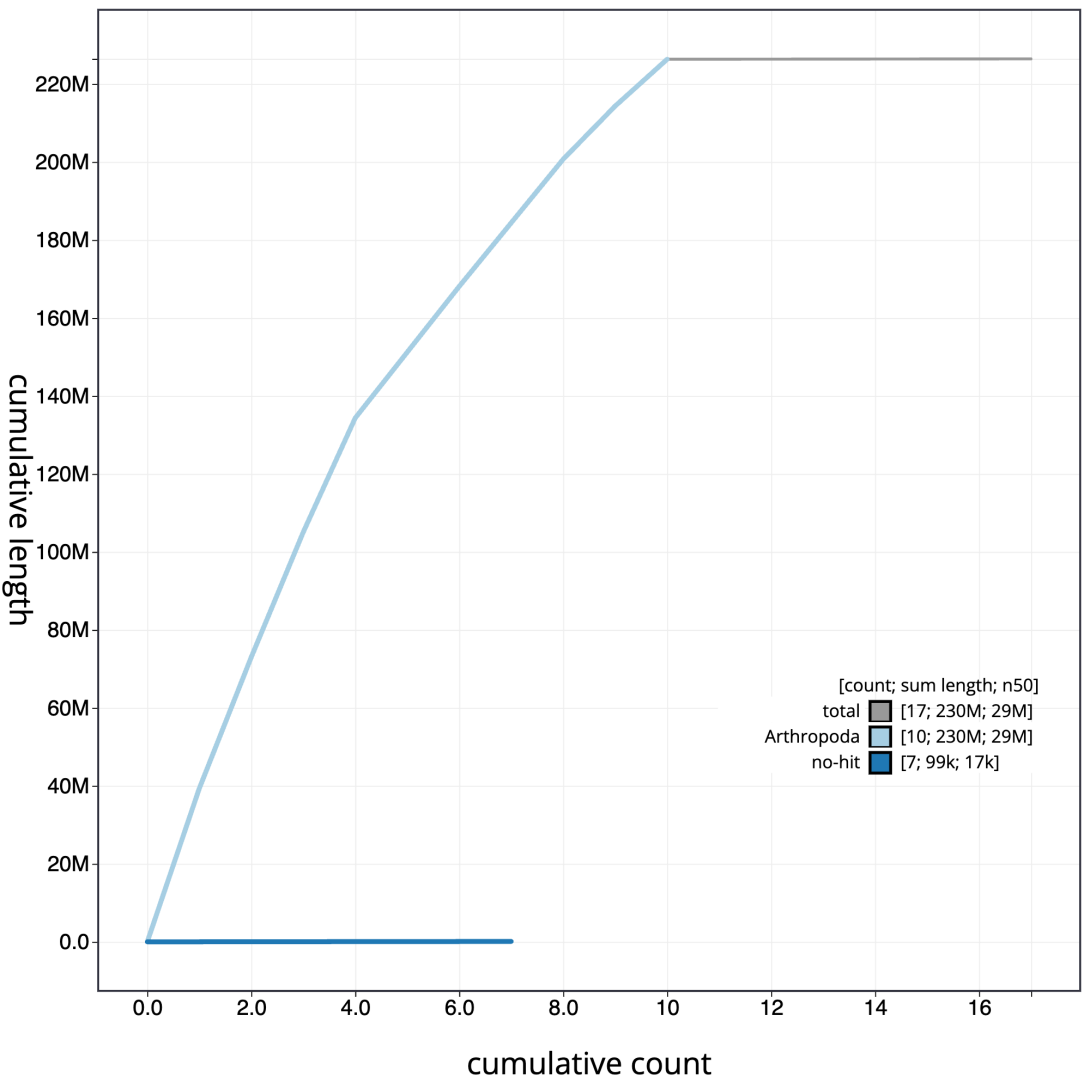
Genome assembly of *Heteropelma amictum*, iyHetAmic1.1: BlobToolKit cumulative sequence plot. The grey line shows cumulative length for all scaffolds. Coloured lines show cumulative lengths of scaffolds assigned to each phylum using the buscogenes taxrule. An interactive version of this figure is available at https://blobtoolkit.genomehubs.org/view/Heteropelma%20amictum/dataset/iyHetAmic1_1/cumulative.

**Figure 5.**
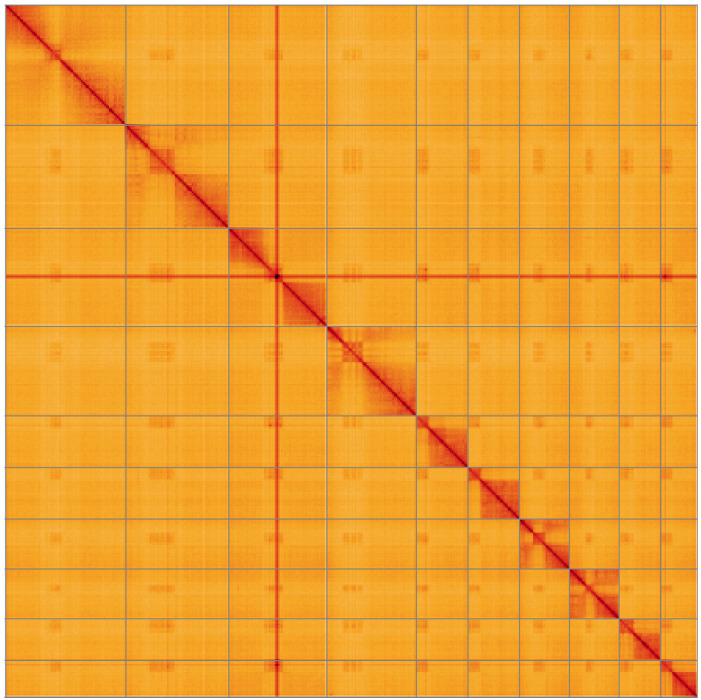
Genome assembly of *Heteropelma amictum*, iyHetAmic1.1: Hi-C contact map of the iyHetAmic1.1 assembly, visualised using HiGlass. Chromosomes are shown in order of size from left to right and top to bottom. An interactive version of this figure may be viewed at https://genome-note-higlass.tol.sanger.ac.uk/l/?d=Kez2u_yFTGaghwgCqkxGEQ.

The final assembly has a total length of 226.4 Mb in 16 sequence scaffolds with a scaffold N50 of 29.3 Mb ( Table 1). The snailplot in Figure 2 provides a summary of the assembly statistics, while the distribution of assembly scaffolds on GC proportion and coverage is shown in Figure 3. The cumulative assembly plot in Figure 4 shows curves for subsets of scaffolds assigned to different phyla. Most (99.95%) of the assembly sequence was assigned to 10 chromosomal-level scaffolds. Chromosome-scale scaffolds confirmed by the Hi-C data are named in order of size ( Figure 5; Table 2). While not fully phased, the assembly deposited is of one haplotype. Contigs corresponding to the second haplotype have also been deposited. The mitochondrial genome was also assembled and can be found as a contig within the multifasta file of the genome submission.

**Table 1.** Genome data for *Heteropelma amictum*, iyHetAmic1.1.

Project accession data		
Assembly identifier	iyHetAmic1.1	
Species	*Heteropelma amictum*	
Specimen	iyHetAmic1	
NCBI taxonomy ID	494771	
BioProject	PRJEB58954	
BioSample ID	SAMEA7701570	
Isolate information	iyHetAmic1, female: abdomen (DNA sequencing), head and thorax (Hi-C data)	
Assembly metrics *		*Benchmark*
Consensus quality (QV)	61.4	*≥ 50*
*k*-mer completeness	100%	*≥ 95%*
BUSCO **	C:93.8%[S:93.7%,D:0.1%], F:1.7%,M:4.5%,n:5,991	*C ≥ 95%*
Percentage of assembly mapped to chromosomes	99.95%	*≥ 95%*
Sex chromosomes	-	*localised homologous pairs*
Organelles	Mitochondrial genome assembled	*complete single alleles*
Raw data accessions		
PacificBiosciences SEQUEL II	ERR10798427	
Hi-C Illumina	ERR10786028	
Genome assembly		
Assembly accession	GCA_959613375.1	
*Accession of alternate haplotype*	GCA_959613325.1	
Span (Mb)	226.4	
Number of contigs	396	
Contig N50 length (Mb)	1.1	
Number of scaffolds	16	
Scaffold N50 length (Mb)	29.3	
Longest scaffold (Mb)	39.3	

* Assembly metric benchmarks are adapted from column VGP-2020 of “Table 1: Proposed standards and metrics for defining genome assembly quality” from ( Rhie *et al.*, 2021). ** BUSCO scores based on the hymenoptera_odb10 BUSCO set using v5.3.2. C = complete [S = single copy, D = duplicated], F = fragmented, M = missing, n = number of orthologues in comparison. A full set of BUSCO scores is available at https://blobtoolkit.genomehubs.org/view/Heteropelma%20amictum/dataset/iyHetAmic1_1/busco.

**Table 2.** Chromosomal pseudomolecules in the genome assembly of *Heteropelma amictum*, iyHetAmic1.

INSDC accession	Chromosome	Length (Mb)	GC%
OY390720.1	1	39.33	40.0
OY390721.1	2	33.68	41.0
OY390722.1	3	32.06	42.0
OY390723.1	4	29.28	40.5
OY390724.1	5	16.91	41.5
OY390725.1	6	16.83	41.5
OY390726.1	7	16.32	42.0
OY390727.1	8	16.3	42.5
OY390728.1	9	13.63	41.5
OY390729.1	10	12.01	42.5
OY390730.1	MT	0.02	18.0

The estimated Quality Value (QV) of the final assembly is 61.4 with *k*-mer completeness of 100%, and the assembly has a BUSCO v5.3.2 completeness of 93.8% (single = 93.7%, duplicated = 0.1%), using the hymenoptera_odb10 reference set ( *n* = 5,991).

Metadata for specimens, barcode results, spectra estimates, sequencing runs, contaminants and pre-curation assembly statistics are given at https://links.tol.sanger.ac.uk/species/494771.

## Methods

### Sample acquisition and nucleic acid extraction

A female *Heteropelma amictum* (specimen ID Ox000709, ToLID iyHetAmic1) was collected from Wytham Woods, Oxfordshire (biological vice-county Berkshire), UK (latitude 51.76, longitude –1.33) on 2020-07-30. The specimen was collected by Liam Crowley (University of Oxford) and identified by Gavin Broad (NHM) and preserved on dry ice.

The workflow for high molecular weight (HMW) DNA extraction at the Wellcome Sanger Institute (WSI) includes a sequence of core procedures: sample preparation; sample homogenisation; DNA extraction; HMW DNA fragmentation; and fragmented DNA clean-up. The iyHetAmic1 sample was weighed and dissected on dry ice with tissue set aside for Hi-C sequencing (as per the protocol at https://dx.doi.org/10.17504/protocols.io.x54v9prmqg3e/v1). For sample homogenisation, the abdomen tissue of the iyHetAmic1 sample was homogenised using a Nippi Powermasher fitted with a BioMasher pestle, following the protocol at https://dx.doi.org/10.17504/protocols.io.5qpvo3r19v4o/v1. HMW DNA was extracted by means of the Automated MagAttract protocol ( https://dx.doi.org/10.17504/protocols.io.kxygx3y4dg8j/v1), in which HMW DNA was sheared into an average fragment size of 12–20 kb in a Megaruptor 3 system with speed setting 30, following the HMW DNA Fragmentation: Diagenode Megaruptor®3 for PacBio HiFi protocol ( https://dx.doi.org/10.17504/protocols.io.8epv5x2zjg1b/v1). Sheared DNA was purified by phase reversible immobilisation (SPRI) ( protocol at https://dx.doi.org/10.17504/protocols.io.kxygx3y1dg8j/v1). In brief, the method employs a 1.8X ratio of AMPure PB beads to sample to eliminate shorter fragments and concentrate the DNA. The concentration of the sheared and purified DNA was assessed using a Nanodrop spectrophotometer and Qubit Fluorometer and Qubit dsDNA High Sensitivity Assay kit. Fragment size distribution was evaluated by running the sample on the FemtoPulse system.

Protocols employed by the Tree of Life laboratory are publicly available on protocols.io: https://dx.doi.org/10.17504/protocols.io.8epv5xxy6g1b/v1.

### Sequencing

Pacific Biosciences HiFi circular consensus DNA sequencing libraries were constructed according to the manufacturers’ instructions. DNA sequencing was performed by the Scientific Operations core at the WSI on a Pacific Biosciences SEQUEL II instrument. Hi-C data were also generated from head thorax tissue of iyHetAmic1 using the Arima2 kit and sequenced on the Illumina NovaSeq 6000 instrument.

### Genome assembly, curation and evaluation

Assembly was carried out with Hifiasm ( Cheng *et al.*, 2021) and haplotypic duplication was identified and removed with purge_dups ( Guan *et al.*, 2020). The assembly was then scaffolded with Hi-C data ( Rao *et al.*, 2014) using YaHS ( Zhou *et al.*, 2023). The assembly was checked for contamination and corrected as described previously ( Howe *et al.*, 2021). Manual curation was performed using HiGlass ( Kerpedjiev *et al.*, 2018) and Pretext ( Harry, 2022). The mitochondrial genome was assembled using MitoHiFi ( Uliano-Silva *et al.*, 2023), which runs MitoFinder ( Allio *et al.*, 2020) or MITOS ( Bernt *et al.*, 2013) and uses these annotations to select the final mitochondrial contig and to ensure the general quality of the sequence.

A Hi-C map for the final assembly was produced using bwa-mem2 ( Vasimuddin *et al.*, 2019) in the Cooler file format ( Abdennur & Mirny, 2020). To assess the assembly metrics, the *k*-mer completeness and QV consensus quality values were calculated in Merqury ( Rhie *et al.*, 2020). This work was done using Nextflow ( Di Tommaso *et al.*, 2017) DSL2 pipelines “sanger-tol/readmapping” ( Surana *et al.*, 2023a) and “sanger-tol/genomenote” ( Surana *et al.*, 2023b). The genome was analysed within the BlobToolKit environment ( Challis *et al.*, 2020) and BUSCO scores ( Manni *et al.*, 2021; Simão *et al.*, 2015) were calculated.

Table 3 contains a list of relevant software tool versions and sources.

**Table 3.** Software tools: versions and sources.

Software tool	Version	Source
BlobToolKit	4.2.1	https://github.com/blobtoolkit/blobtoolkit
BUSCO	5.3.2	https://gitlab.com/ezlab/busco
Hifiasm	0.16.1-r375	https://github.com/chhylp123/hifiasm
HiGlass	1.11.6	https://github.com/higlass/higlass
Merqury	MerquryFK	https://github.com/thegenemyers/MERQURY.FK
MitoHiFi	2	https://github.com/marcelauliano/MitoHiFi
PretextView	0.2	https://github.com/wtsi-hpag/PretextView
purge_dups	1.2.3	https://github.com/dfguan/purge_dups
sanger-tol/genomenote	v1.0	https://github.com/sanger-tol/genomenote
sanger-tol/readmapping	1.1.0	https://github.com/sanger-tol/readmapping/tree/1.1.0
YaHS	1.2a	https://github.com/c-zhou/yahs

### Wellcome Sanger Institute – Legal and Governance

The materials that have contributed to this genome note have been supplied by a Darwin Tree of Life Partner. The submission of materials by a Darwin Tree of Life Partner is subject to the **‘Darwin Tree of Life Project Sampling Code of Practice’**, which can be found in full on the Darwin Tree of Life website here. By agreeing with and signing up to the Sampling Code of Practice, the Darwin Tree of Life Partner agrees they will meet the legal and ethical requirements and standards set out within this document in respect of all samples acquired for, and supplied to, the Darwin Tree of Life Project. 

Further, the Wellcome Sanger Institute employs a process whereby due diligence is carried out proportionate to the nature of the materials themselves, and the circumstances under which they have been/are to be collected and provided for use. The purpose of this is to address and mitigate any potential legal and/or ethical implications of receipt and use of the materials as part of the research project, and to ensure that in doing so we align with best practice wherever possible. The overarching areas of consideration are:

•   Ethical review of provenance and sourcing of the material

•   Legality of collection, transfer and use (national and international) 

Each transfer of samples is further undertaken according to a Research Collaboration Agreement or Material Transfer Agreement entered into by the Darwin Tree of Life Partner, Genome Research Limited (operating as the Wellcome Sanger Institute), and in some circumstances other Darwin Tree of Life collaborators.

## Data Availability

European Nucleotide Archive: *Heteropelma amictum*. Accession number PRJEB58954; https://identifiers.org/ena.embl/PRJEB58954 ( Wellcome Sanger Institute, 2023). The genome sequence is released openly for reuse. The *Heteropelma amictum* genome sequencing initiative is part of the Darwin Tree of Life (DToL) project. All raw sequence data and the assembly have been deposited in INSDC databases. The genome will be annotated using available RNA-Seq data and presented through the Ensembl pipeline at the European Bioinformatics Institute. Raw data and assembly accession identifiers are reported in Table 1.

## Author information

Members of the University of Oxford and Wytham Woods Genome Acquisition Lab are listed here: https://doi.org/10.5281/zenodo.7125292.

Members of the Darwin Tree of Life Barcoding collective are listed here: https://doi.org/10.5281/zenodo.4893703.

Members of the Wellcome Sanger Institute Tree of Life programme are listed here: https://doi.org/10.5281/zenodo.4783585.

Members of Wellcome Sanger Institute Scientific Operations: DNA Pipelines collective are listed here: https://doi.org/10.5281/zenodo.4790455.

Members of the Tree of Life Core Informatics collective are listed here: https://doi.org/10.5281/zenodo.5013541.

Members of the Darwin Tree of Life Consortium are listed here: https://doi.org/10.5281/zenodo.4783558.

## References

[ref-1] Abdennur N Mirny LA : Cooler: Scalable storage for Hi-C data and other genomically labeled arrays. *Bioinformatics.* 2020;36(1):311–316. 10.1093/bioinformatics/btz540 31290943 PMC8205516

[ref-2] Allio R Schomaker-Bastos A Romiguier J : MitoFinder: Efficient automated large-scale extraction of mitogenomic data in target enrichment phylogenomics. *Mol Ecol Resour.* 2020;20(4):892–905. 10.1111/1755-0998.13160 32243090 PMC7497042

[ref-3] Bernt M Donath A Jühling F : MITOS: Improved *de novo* metazoan mitochondrial genome annotation. *Mol Phylogenet Evol.* 2013;69(2):313–319. 10.1016/j.ympev.2012.08.023 22982435

[ref-4] Challis R Richards E Rajan J : BlobToolKit - interactive quality assessment of genome assemblies. *G3 (Bethesda).* 2020;10(4):1361–1374. 10.1534/g3.119.400908 32071071 PMC7144090

[ref-30] Cheng H Concepcion GT Feng X : Haplotype-resolved *de novo* assembly using phased assembly graphs with hifiasm. *Nat Methods.* 2021;18(2):170–175. 10.1038/s41592-020-01056-5 33526886 PMC7961889

[ref-6] Di Tommaso P Chatzou M Floden EW : Nextflow enables reproducible computational workflows. *Nat Biotechnol.* 2017;35(4):316–319. 10.1038/nbt.3820 28398311

[ref-5] Gauld ID : The taxonomy of the genus *Heteropelma* Wesmael (Hymenoptera: Ichneumonidae). *Bulletin of the British Museum (Natural History). Entomology.* 1976;34(3):153–219. Reference Source

[ref-9] Gauld ID Mitchell PA : Ichneumonidae. Orthopelmatinae and Anomaloninae. *Handbooks for the Identification of British Insects.* 1977;8(2):1–32.

[ref-8] Guan D McCarthy SA Wood J : Identifying and removing haplotypic duplication in primary genome assemblies. *Bioinformatics.* 2020;36(9):2896–2898. 10.1093/bioinformatics/btaa025 31971576 PMC7203741

[ref-31] Harry E : PretextView (Paired REad TEXTure Viewer): A desktop application for viewing pretext contact maps. 2022; [Accessed 19 October 2022]. Reference Source

[ref-10] Howe K Chow W Collins J : Significantly improving the quality of genome assemblies through curation. *GigaScience.* Oxford University Press,2021;10(1): giaa153. 10.1093/gigascience/giaa153 33420778 PMC7794651

[ref-11] Kerpedjiev P Abdennur N Lekschas F : HiGlass: web-based visual exploration and analysis of genome interaction maps. *Genome Biol.* 2018;19(1): 125. 10.1186/s13059-018-1486-1 30143029 PMC6109259

[ref-12] Manni M Berkeley MR Seppey M : BUSCO update: Novel and streamlined workflows along with broader and deeper phylogenetic coverage for scoring of eukaryotic, prokaryotic, and viral genomes. *Mol Biol Evol.* 2021;38(10):4647–4654. 10.1093/molbev/msab199 34320186 PMC8476166

[ref-13] Pénigot W : Liste et clé d’identification pour les Anomaloninae de la faune de France, avec la description d’une espèce nouvelle du genre Therion (Hymenoptera, Ichneumonidae). *Bulletin de La Société Entomologique de France.* 2021;126:253–328. 10.32475/bsef_2182

[ref-14] Rao SSP Huntley MH Durand NC : A 3D map of the human genome at kilobase resolution reveals principles of chromatin looping. *Cell.* 2014;159(7):1665–1680. 10.1016/j.cell.2014.11.021 25497547 PMC5635824

[ref-15] Rhie A McCarthy SA Fedrigo O : Towards complete and error-free genome assemblies of all vertebrate species. *Nature.* 2021;592(7856):737–746. 10.1038/s41586-021-03451-0 33911273 PMC8081667

[ref-16] Rhie A Walenz BP Koren S : Merqury: Reference-free quality, completeness, and phasing assessment for genome assemblies. *Genome Biol.* 2020;21(1): 245. 10.1186/s13059-020-02134-9 32928274 PMC7488777

[ref-18] Rosenberg HT : The biology and distribution in France of the larval parasites of *Cydia pomonella,* L. *B Entomol Res.* 1934;25(2):201–256. 10.1017/S0007485300012657

[ref-17] Simão FA Waterhouse RM Ioannidis P : BUSCO: assessing genome assembly and annotation completeness with single-copy orthologs. *Bioinformatics.* 2015;31(19):3210–3212. 10.1093/bioinformatics/btv351 26059717

[ref-19] Surana P Muffato M Qi G : sanger-tol/readmapping: sanger-tol/readmapping v1.1.0 - Hebridean Black (1.1.0). *Zenodo.* 2023a; [Accessed 21 July 2023]. 10.5281/zenodo.7755665

[ref-20] Surana P Muffato M Sadasivan Baby C : sanger-tol/genomenote (v1.0.dev). *Zenodo.* 2023b; [Accessed 21 July 2023]. 10.5281/zenodo.6785935

[ref-24] Tothill JD : The natural control of the Fall Webworm ( *Hyphantria cunea* Drury). With an account of its several parasites. *Canadian Department of Agriculture Bulletin Entomological Bulletin.* 1922;19:1–107.

[ref-21] Uliano-Silva M Ferreira JGRN Krasheninnikova K : MitoHiFi: a python pipeline for mitochondrial genome assembly from PacBio high fidelity reads. *BMC Bioinformatics.* 2023;24(1): 288. 10.1186/s12859-023-05385-y 37464285 PMC10354987

[ref-22] Vasimuddin M Misra S Li H : Efficient Architecture-Aware Acceleration of BWA-MEM for Multicore Systems.In: *2019 IEEE International Parallel and Distributed Processing Symposium (IPDPS).*IEEE,2019;314–324. 10.1109/IPDPS.2019.00041

[ref-32] Wellcome Sanger Institute: The genome sequence of an ichneumonid wasp, *Heteropelma amictum* (Fabricius, 1775). European Nucleotide Archive.[dataset], accession number PRJEB58954,2023. 10.12688/wellcomeopenres.20364.1 PMC10905020 38434733

[ref-23] Zhou C McCarthy SA Durbin R : YaHS: yet another Hi-C scaffolding tool. *Bioinformatics.* 2023;39(1): btac808. 10.1093/bioinformatics/btac808 36525368 PMC9848053

