# Supplementary material for: The genome sequence of an ichneumonid wasp, Heteropelma amictum (Fabricius, 1775)
Source: Wellcome Open Res. 2023 Nov 20;8:537. [Version 1] doi: 10.12688/wellcomeopenres.20364.1 (PMC10905020; doi:10.12688/wellcomeopenres.20364.1)
Supplement: Peer review history

# Reviewer report: Reviewer response for version 1

**DOI:** 10.21956/wellcomeopenres.22550.r74444

**Published:** 2024-02-28

**Authors:** Jerome Hui

**Affiliations:** 1 The Chinese University of Hong Kong, Hong Kong, Hong Kong

Crowley and colleagues report the genome sequence of wasp *Heteropelma amictum *(Fabricius 1775). This species can be found in many places in Britain. Molecular data of this species are mainly confined to COI and ribosomal sequences (as well as transcriptomes) deposited to the NCBI database. This new genome resource is important and will be very useful for further studies, such as understanding the ecological, evolutionary, and genomics questions related to insects more widely.

 This genome resource is excellent from the summary statistics, with good BUSCO numbers, high sequence continuity (scaffold N50), and majority of sequences contained on the 10 pseudochromosomes (plus mitochondrion). To sum up, this is another valuable contribution from the Darwin Tree of Life Consortium.

Are sufficient details of methods and materials provided to allow replication by others?

Yes

Is the rationale for creating the dataset(s) clearly described?

Yes

Are the datasets clearly presented in a useable and accessible format?

Yes

Are the protocols appropriate and is the work technically sound?

Yes

Reviewer Expertise:

Genomics, evolution, invertebrates

I confirm that I have read this submission and believe that I have an appropriate level of expertise to confirm that it is of an acceptable scientific standard.

# Reviewer report: Reviewer response for version 1

**DOI:** 10.21956/wellcomeopenres.22550.r72009

**Published:** 2024-02-07

**Authors:** Tilman Schell

**Affiliations:** 1 LOEWE Centre for Translational Biodiversity Genomics (LOEWE-TBG), Frankfurt am Main, Germany

Review of

 The genome sequence of an ichneumonid wasp, Heteropelma amictum (Fabricius, 1775) from L. M. Crowley and G. R. Broad

 Crowley and Broad present a data note on the chromosome level genome assembly of Heteroplema amictum. As for many other of DToL's data notes, the underlying raw data is sufficient and of high quality, methods are described in a way to ensure reproducibility and results are very good.

 In general, there are only a few minor remarks.

1. In the first sentence of the Background paragraph, you write "is one of our more". Despite being written from a British perspective, I would recommend rephrasing "our" to something more general (e.g. "one of Britain's most" or simply "one of the most").
2. Not sure if this will be fixed during production but Figure 2 has low resolution and looks not ideal.
3. In the contact map (Figure 5) there is one region in the middle of the third largest scaffold, which shows signal to all regions in the assembly (red cross). Do you know what caused this? Is this a technical artifact?

Are sufficient details of methods and materials provided to allow replication by others?

Yes

Is the rationale for creating the dataset(s) clearly described?

Yes

Are the datasets clearly presented in a useable and accessible format?

Yes

Are the protocols appropriate and is the work technically sound?

Yes

Reviewer Expertise:

Genome assembly, Bioinformatics

I confirm that I have read this submission and believe that I have an appropriate level of expertise to confirm that it is of an acceptable scientific standard.
